# Fungal association with sessile marine invertebrates

**DOI:** 10.3389/fmicb.2014.00228

**Published:** 2014-05-15

**Authors:** Oded Yarden

**Affiliations:** Department of Plant Pathology and Microbiology, The Robert H. Smith Faculty of Agriculture, Food and Environment, The Hebrew University of JerusalemRehovot, Israel

**Keywords:** marine fungi, ascidian, marine sponge, coral health

## Abstract

The presence and association of fungi with sessile marine animals such as coral and sponges has been well established, yet information on the extent of diversity of the associated fungi is still in its infancy. Culture – as well as metagenomic – and transcriptomic-based analyses have shown that fungal presence in association with these animals can be dynamic and can include “core” residents as well as shifts in fungal communities. Evidence for detrimental and beneficial interactions between fungi and their marine hosts is accumulating and current challenges include the elucidation of the chemical and cellular crosstalk between fungi and their associates within the holobionts. The ecological function of fungi in association with sessile marine animals is complex and is founded on a combination of factors such as fungal origin, host health, environmental conditions and the presence of other resident or invasive microorganisms in the host. Based on evidence from the much more studied terrestrial systems, the evaluation of marine animal–fungal symbioses under varying environmental conditions may well prove to be critical in predicting ecosystem response to global change, including effects on the health of sessile marine animals.

## INTRODUCTION

There is no consensus on the definition of marine fungi, even though it is clear that the grouping of marine fungi is primarily based on an ecological rather than a taxonomical basis (Kohlmeyer and Kohlmeyer, 1979; Hyde et al., 2000). Commonly used descriptions include “marine-derived” or “marine-associated” fungi, yet “facultative-marine” and “obligate-marine” would, most likely, best distinguish between fungi isolated from marine niches versus those requiring the marine environment. Much of the research on these organisms has focused on fungi from marine environments such as obtained or found associated with mangroves, wood substrates, sediments (Jones, 2011b and references within), as well as on fungal infections of marine mammals (Higgins, 2000).

The occurrence of fungal associations with other organisms within the marine environment has been reported and discussed for over a century, including the concerns whether fungi can, at all, grow in sea water (Murray, 1893). One of the earliest reports on actual parasitism by a marine fungus (albeit on an algae) was documented that same year by Church (1893). A significant landmark in the study of fungi from the marine environment was the report by Barghoorn and Linder (1944) who in addition to their descriptions of marine-derived fungi stated that “The fact that a score or more of species have been described as occurring in the sea is of importance since it shows that fungi not only tolerate salt water, but indeed that marine conditions furnish a normal habitat for the relatively small number of fungi that have become adapted to it.” Since then, the number of fungal species (over 800) described from marine environments and the rate at which they are currently being described indicates that the marine fungal community is larger than originally considered (Jones et al., 2009; Jones, 2011a,b). Nonetheless, whereas the *Symbiodinium* and marine prokaryotic and viral communities and their possible contributions to the niches they reside in received increased attention during the last decades (e.g., Breitbart, 2012; Garren and Azam, 2012; Weber and Medina, 2012; Moitinho-Silva et al., 2014), our current understanding of the fungal communities in marine environments is still extremely limited (Amend et al., 2012). The fungal kingdom is estimated to be comprised of approximately 1.5 million species, with less than 10% of them described to date (Hawksworth, 2001). As the identification of fungi associated with sessile marine animals and the realization of the ecological significance they may have is fairly recent, it is highly likely that many new species (some with unique attributes/ecological roles) within these niches have yet to be isolated/identified.

In this short review, evidence accumulated for the presence and association of fungi with corals, sponges, and ascidians will be provided as well as discussion of the potential impact fungi may prove to have in these niches.

## CORALS

The phylum Cnidaria, comprised of an approximate 10,000 species (Zhang, 2011) has been the most widely studied with regard to fungal prevalence. While the presence of fungi in coral hosts is acknowledged in the literature (Kendrick et al., 1982; Le Campion-Alsumard et al., 1995; Bentis et al., 2000; Golubic et al., 2005), insight as to the mechanistic nature of the interactions between the two is scarce.

In an in-depth SSU-based amplicon sequencing/transcriptomic analysis of the fungal community associated with the coral *Acropora hyacinthus*, the authors determined the presence of a diverse, metabolically active, fungal community (Amend et al., 2012). Interestingly, they have also uncovered a phylogenetically diverse core assemblage of fungi consistently associated with *A. hyacinthus*. The community was correlated with the host rather than differences in environment (e.g., water temperature) or the presence of *Symbiodinium* partners in the sample (Amend et al., 2012). Metagenomic analysis of bleaching *A. millepora* revealed a 3-fold increase in fungi-like sequences, yet the role of fungi during this stress is unclear (Littman et al., 2011). In analysis of the *Porites astreoides* metagenome, fungi were found to be highly prevalent and have been implied to play a possible role in at least two processes of the nitrogen cycle within the coral, suggesting a positive role of fungi, along with other associated microorganisms within the holobiont (Wegley et al., 2007).

Recent incorporation of transcriptome-based analyses have also provided evidence for the presence of fungal species in cnidarians, such as in the case of the sea anemone *Aiptasia* in which changes in the microbial transcriptome have been monitored under conditions in which dinoflagellates were present versus aposymbiotic (bleached) states (Lehnert et al., 2014). In what way these changes influence the coral is not yet clear, yet given the fact that some coral (like members of the temperate genus *Astrangia*) go through seasonal changes in algal presence/activity (Dimond and Carrington, 2007), fluctuations in the presence of fungi or their activity may well be expected to accompany or be involved in seasonal or environmental changes. In addition to the variations in fungal community composition or activity, altered spatial distribution of core/resident fungi may also occur, as has been described in *A. formosa,* where fungi resident in healthy tissue of the coral proliferate into the skeletal cavity in stressed animals (Yarden et al., 2007). An additional form of interaction may involve a succession in biotic/abiotic factors involved in conferring a disease or syndrome. Such is the case described for black band and density-banding of *Porites* spp., where following an increase in the abundance of endolithic algae, these are attacked by fungi, which proliferate and are associated with production of the dark pigment (Priess et al., 2000). Hence, the combinations of temporal and spatial presence of fungi, along with their activities within the host appear to be dynamic and can clearly impact coral health. Determining the kinetics of fungal association with the host and other holobiont constituents is a key to the eventual understanding of their ecological significance.

Endolithic fungi have been shown to elicit a defensive response in corals, and have thus been suggested to elicit a parasitic, rather than saprophytic (or mutualistic) nature of association (Le Campion-Alsumard et al., 1995; Bentis et al., 2000; Golubic et al., 2005; Raghukumar and Ravindran, 2012). Perhaps the most striking example for potential parasitism has been the study of *Aspergillus sydowii*, a common terrestrial fungus suggested to cause an epidemic in the sea fan *Gorgonia ventalina* that can also confer symptoms to *G. flabellum* (Geiser et al., 1998; Kim and Harvell, 2004). The ecology and host response of sea fan coral Aspergillosis has been recently reviewed by Burge et al. (2013). The disease causing agent was suggested to be introduced as dust-borne propagules originating from the Sahara (Garrison et al., 2003). However, evidence of panmixia and lack of isolation by distance, along with high genetic diversity of the *A. sydowii* isolates analyzed and lack of evidence supporting the presence of the fungus in sampled African dust suggest that a single origin of the pathogen or a limited number of introductions is probably not the basis of Caribbean sea fan Aspergillosis (Rypien, 2008; Rypien et al., 2008). Furthermore, the presence of additional opportunistic fungi associated with sea fans (including sea fans in the Pacific; Barrero-Canosa et al., 2013) suggests that Aspergillosis, and related diseases, may be caused by more than one fungal species. A changing environment may also affect the nature of associations, via the status of the holobiont, as some coral species have been shown to exhibit a temperature-dependent immune response, including changes in melanin production, circulating amoebocytes and antioxidant levels (Mydlarz et al., 2010). These data imply that the potential detrimental (or beneficial) effects in which fungi are involved may be more complex than originally perceived and are based on a combination of factors such as pathogen origin, host health, environmental conditions and the presence of other resident or invasive microorganisms in the host. Furthermore, it is highly conceivable that some fungi may alter their function (e.g., from commensals to opportunists/pathogens) as a result of changes in environmental conditions, host vigor or holobiont composition/activity.

Taken together, most studies performed on coral-associated fungi have focused on either parasitic or opportunistic interactions. Even though the majority of these describe either the fungal species associated with coral or potential detrimental outcomes of fungal presence within the animals, the importance of another key form of interactions – mutualism, has yet to be probed in detail, even though support for this notion has been clearly discussed (Wegley et al., 2007). Evidence from plant and terrestrial animals suggest that it will not be surprising to find such interactions in coral (and other sessile marine animals), yet the actual nature of mutual relationships have yet to be substantiated.

## SPONGES

It is not surprising to find fungi associated with sponges, along with a plethora of other microorganisms (Hentschel et al., 2012). The fact that 40–60% of the sponge biomass is comprised of associated microorganisms, along with the nature of sponge feeding, based on the filtering of vast volumes of water (known to harbor fungal propagules), provides a high potential for the presence of fungi. Over 20 years ago Morrison-Gardiner (2002) isolated over a hundred fungal strains from marine sponges. Höller et al. (2000) isolated fungi from 16 species of sponges from tropical, subtropical, and temperate waters. In both cases, the fungi belonged to both marine and ubiquitous genera and produced diverse metabolites, many of which were previously unknown. Eighty five fungal taxa have been isolated from the Mediterranean Sea sponge *Ircinia variabilis* (formerly *Psammocinia* sp.), mainly by using a “sample compressing” method, in combination with fungicides-amended media (Paz et al., 2010). Here, too, abundant “terrestrial” taxa such as *Acremonium* and *Penicillium* were found, along with previously undescribed *Phoma* and *Trichoderma* species. Interestingly, when some of the *Trichoderma* spp. were examined, a significant number of the analyzed strains were shown to be halo-sensitive, suggesting that not the entire community was preferentially adapted to the marine environment and perhaps some of them were recently introduced or acquired from terrestrial sources (run-off or air-borne; Gal-Hemed et al., 2011).

Overall, the extent and ecological nature of fungal–sponge associations remains to be determined. Evidence for specificity of fungal communities within different sponges has been provided in the case of *Suberites zeteki* and *Mycale armata* (Gao et al., 2008; Li and Wang, 2009), yet to what extent fungal community signatures are hallmarks of different sponge microbiomes will need to be further established once additional data is collected. Based on metagenomic and gene enrichment analysis of the deep sea sponge *Lamellomorpha* sp., Li et al. (2014) proposed that eukaryotic symbionts, including fungi, along with their prokaryotic counterparts, can exhibit different metabolic potentials, especially in nitrogen and carbon metabolism. Furthermore, genetic interactions among them may also occur. Interestingly, even though fungi have been isolated from sponges, contrary to corals, no evidence for the presence of hyphal structures within sponges has surfaced over the years despite in-depth ultrastructural analyses carried out on a wide range of sponges (Maldonado et al., 2005). This raises the possibility that fungal propagules (presumably sexual or asexual spores, along with hyphal fragments) do not germinate to produce the standard hyphal cells but rather adapt to direct spore production in the liquid environment (Martinelli, 1976). This, of course, does not refer to unicellular yeasts, which under most circumstances do not produce hyphae or hyphae-like structures. In fact, based on TEM analysis, Maldonado et al. (2005) described the vertical transmission of a unicellular fungus via the oocytes in *Chondrilla nucula*, further substantiating the close potential interaction between fungus and sponge.

In contrast to coral, assessment of disease causing microbial agents in sponges appears to be more difficult, perhaps due to the complexity of the microbial communities associated with sponges (Webster and Taylor, 2012). However, the potential of a sponge to be a symptomless carrier of a coral disease-causing agent has been suggested, following the isolation of a sea fan-infectious strain of *A. sydowii* from *Spongia obscura* (Ein-Gil et al., 2009). Whether some fungi may prove to be omnivorous opportunists or pathogens raises the question concerning fungi as potential threats to more than one sessile marine species.

## ASCIDIANS

To date, only few reports describing fungal association with ascidians have been published and most analyses have focused on prokaryotic symbionts of these animals (Blasiak et al., 2014; Erwin et al., 2014). Perhaps the most detailed analysis of fungal diversity in ascidians has been reported by Menezes et al. (2010), who isolated representatives of over 15 fungal genera (the most abundantly described were *Trichoderma*, *Phoma,* and *Cladosporium* spp.) cultured from *Didemnum* spp. In earlier studies, Morrison-Gardiner (2002) described over two dozen fungal strains isolated from various chordates, the majority of which were from the genera *Aspergillus* and *Cladosporium*. At least one case of an acidian-associated fungus (*Camarosporium* sp.) was included in that report. Other reports, mainly related to bioprospecting for novel natural compounds include representatives of known fungal genera such as *Penicillium* in the case of isocoumarin derivatives (Xin et al., 2007) and *Aspergillus* strains that produce cytotoxic compounds, isolated from *Eudistoma vannamei* (Montenegro et al., 2012). In addition, a likely new species of the family Diatrypaceae was isolated from an unnamed ascidian in the Bahamas (Oh et al., 2005, 2010). Co-culturing this fungus with a fungal-associated marine bacterium induced a fungal diterpene biosynthesis pathway, suggesting a potential fungal-bacterial interaction which could result in the production of secondary metabolites. Whether or not this co-metabolic process occurs in in the natural environment is not known.

## ABUNDANT BIOACTIVE COMPOUNDS IN THE HOLOBIONT MAY HAVE ROLES IN HOST-FUNGAL AND FUNGAL-MICROBE INTERACTIONS

Most of the emphases in the study of sessile marine animal–fungal interactions have been dedicated to the outcome of such interactions in respect to the hosts and by far less on the tolerance/susceptibility and physiological adaptation of the fungi involved. Chemical defense mechanisms have been considered as part and parcel of the success and survival strategies evolved in many sessile marine invertebrates. It is thus highly probable that some of the chemicals produced are directly involved in determining the composition and activity of resident and introduced microbiota (as well as other potential predators or invaders). The antifungal nature of some of the chemicals produced by such animals and their accompanying microflora can provide a potentially hostile environment for fungi (Donia and Hamann, 2003; Wang et al., 2013 and references within). It is not always clear which holobiont constituent (or their combinations) produce many of the compounds described. Nonetheless, cultured fungi isolated from sessile marine animals have been demonstrated to be capable of producing novel chemicals which have the potential to affect the marine hosts and their microbiome. These findings have been driven both by ecological/physiological-based research as well as bioprospecting efforts aimed at obtaining novel bioactive compounds (e.g., Höller et al., 2000; Wang et al., 2008; Cohen et al., 2011; Panizel et al., 2013 and reviewed in Raghukumar, 2008; Imhoff et al., 2011; Rateb and Ebel, 2011). Though understanding the nature of the cross-talk between the host and its associated microorganisms has mainly focused on the bacterial components, some studies on host–fungal interactions have been carried out. Wang et al. (2013) reviewed some of the chemical defense arsenal produced in sponges. This includes a broad range of compounds (from amino acid derivatives to nucleosides, macrolides, porphyrins, terpenoids, aliphatic cyclic peroxides, and sterols, just to mention a few groups) that have been shown to exhibit biological activity on a variety of cell types (sponges and others). Anti-microbial chemicals are also produced within sponges, and have been shown to specifically inhibit some Gram-positive or Gram-negative bacteria as well as fungi. An example of the potential sponge chemical response to fungi was reported by Ward et al. (2007) who showed that extracts from *A. sydowii*-infected *G. ventalina* inhibited the fungus significantly more than those from uninfected coral. Furthermore, these extracts exhibited increased potency when obtained from sea fans grown at elevated temperatures (in which higher severity of disease was also observed). Perović-Ottstadt et al. (2004) described a cell surface receptor that recognizes (1 → 3)-β-D-glucans (a fungal cell wall component) in the demosponge *S. domuncula* and that this is followed by a transduction of signals which include tyrosine protein phosphorylation. Such a recognition event can lead to exclusion/killing of the fungus or, conversely, the establishment of a symbiotic interaction. Regardless, the richness of the microbial community associated with sponges is clear evidence for the discriminatory function of some of the chemicals and corresponding cellular reactions produced.

Understanding of the effects of antifungal compounds (and other responses) produced by sessile marine holobionts and the role of fungal-produced compounds warrants additional research. This includes, in addition to chemical structure elucidation, the mode of anti-fungal action and the cellular signals involved both in the host and the fungus. Perhaps most challenging of all, is determining the significance/ecological roles of these compounds in the holobiont under varying environmental conditions.

## DO FUNGI HAVE THE POTENTIAL OF ALTERING THE RESPONSE OF SESSILE MARINE ANIMALS TO GLOBAL CHANGE?

Documentation concerning global climate changes and their possible effects on marine animals is accumulating (Harvell et al., 2002; Hoegh-Guldberg and Bruno, 2010). However, little is known about the possible changes/effects they may have on the fungi associated with these animals and on their possible involvement or role in the consequences of a changing environment. The effect of global changes on plant performance in the case of fungal pathogens is, by far, better documented than in the marine system. Kivlin et al. (2013) performed a meta-analysis of publications on the indirect responses to global changes (including enriched CO_2_ levels and warming) of plants associated with four classes (leaf, arbuscular mycorrhizal fungi, ectomycorrhizal fungi, and dark septate fungi) of endophytes (fungi residing within the host for at least part of their life cycle without causing apparent detrimental effects). For the fungal groups analyzed, presence of the symbionts did not significantly influence the effect of CO_2_ on host plant performance. Warming had a differential effect, based on the symbiont class. An increase in temperature can promote plant growth and in the case of the presence of beneficial endophytes such an increase can further enhance plant biomass accumulation. Adversely, the presence of a potentially detrimental endophyte (such as those belonging to the *Phialocephala fortinii* s.l. – *Acephala applanata* species complex) can result in a reduction in plant biomass accumulation under elevated temperature conditions. The changes analyzed involve an array of factors ranging from the genetic background of the host to the colonization capacity of the symbiont as well as interactions with other fungal strains/taxa (Reininger et al., 2012). To what extent the interactions in the marine niches in terms of the combined genetic backgrounds of sessile marine animal hosts and the fungi involved, along with environmental changes, are analogous to those described in some of the terrestrial systems, has yet to be analyzed. The study of a few aspects of such interactions has been initiated as stated in some of the examples above (Ward et al., 2007; Amend et al., 2012) and some marine-derived fungi have been shown to alter their growth rates as a result of combined changes in salinity and temperature (Lorenz and Molitoris, 1992).

Oceanic pH levels are predicted to decrease by.0.4 units within the current century, due to increases in atmospheric CO_2_ concentrations (Caldeira and Wickett, 2005; Hoegh-Guldberg et al., 2007). Meron et al. (2012) reported no significant changes in coral-associated bacterial communities along a natural pH gradient in the vicinity of volcanic vents, yet fungi were not monitored in that study. Nonetheless, under controlled conditions, a marked increase in the fungal community was observed under stress conditions in the coral *P. compressa* (Thurber et al., 2009). Krause et al. (2013) have used a microcosm experimental system to follow the changes in fungal colony forming units (CFUs) under altered pH conditions and found significant increases (9-fold) in fungal CFUs when pH levels were reduced to 7.82, when compared to the ambient 8.0 levels present in some North Sea locations. These data suggest that dissemination and possible proliferation of some fungi may increase as a result of acidification of the marine environment. Should they prove to affect holobiont health, the evaluation of marine animal–fungal symbioses under varying environmental conditions may well prove to be critical in predicting ecosystem resilience to global change.

## SESSILE MARINE ANIMAL-ASSOCIATED FUNGI – A THREAT?

Fisher et al. (2012) listed several cases where fungi were not only shown to be threats to plant/animal health but have also caused severe die-offs and extinctions in wild species and can impact biodiversity in a manner affecting food security and ecosystem health. In terrestrial plant systems, negative density dependence has been suggested as a mechanism that promotes community diversity on the basis of preventing competitive exclusion of some species by the more dominant ones [The Janzen–Connell hypothesis (Janzen, 1970; Connell, 1971)]. Recently, Bagchi et al. (2014) have experimentally tested the impact of fungi on plant diversity and have shown that fungicide treatment reduced the effective number of plant species by approximately 16%, implicating a role pathogenic fungi have in promoting diversity within the seedling community analyzed. The fundamentals of the Janzen–Connell model describing the promotion of diversity have been shown to be valid in an aquatic ecosystem. Marhaver et al. (2013) demonstrated that survivorship of *Orbicella faveolata* (formerly *Montastraea faveolata*) planulae was not only dependent on their proximity to adults of other taxa, but also significantly reduced in water collected near conspecific adults when compared to sterile water. The latter data support the prediction that mortality is due to the activity of host-specific detrimental microorganisms. Whether or not fungi are involved in the described mortality and if such occurrences are relevant to coral that reproduce mainly by fragmenting (or other sessile animals) has yet to be determined, yet the foundations for examining the model with regard to fungal activity have been set.

In conclusion, fungi have gained recognition as both resident and functional components of sessile marine animal microbiomes. The extent of their diversity and function has only begun to be revealed and determining their contributions and effects on the health, maintenance, survival and proliferation of sessile marine animals is a timely task, especially under a changing environment. It is tempting to try and extrapolate from terrestrial to marine environments. One can speculate that fungi may well contribute to sessile marine animal communities in related ways and that changes in the prevalence of fungi associated with marine species may have significant effects on maintaining the biodiversity of the latters. Based on the vast evidence on the complexity and diversity of plant-microbe interactions, we can expect an equivalently rich variety of relationships in sessile organisms of the marine environment.

## Conflict of Interest Statement

The author declares that the research was conducted in the absence of any commercial or financial relationships that could be construed as a potential conflict of interest.
